# Fungal positivity seen in tertiary care hospital during COVID-19 pandemic

**DOI:** 10.1099/acmi.0.000640.v5

**Published:** 2025-01-29

**Authors:** Ravinder Kaur, Deepti Rawat, Ashish William, Pradeep Kumar Singh, Neelam S.S. Kandir, Akanksha Sharma

**Affiliations:** 1 Department of Microbiology, Lady Hardinge Medical College & Associated Hospitals, New Delhi, Delhi, India

**Keywords:** candidiasis, aspergillosis, mucormycosis, CAM (Covid-19-associated mucormycosis)

## Abstract

Coronavirus disease 2019 (COVID-19) pandemic has been prevailing for more than a year, associated with an increased number of opportunistic invasive fungal infections in patients who have been critically ill or immunocompromised. In this retrospective study, details of various clinical specimens received from suspected patients of fungal infections were studied. Fungal cultures were positive in 64% (51 out of 79) of COVID-19-positive patients and 43% (163 out of 381) of COVID-19-negative patients during the second wave of COVID-19 in 2021. Among COVID-19-infected patients, the most commonly isolated fungi were *Candida* spp. (63%), followed by *Aspergillus* spp. (15%) and *Mucor* spp. (6%). The majority of samples that tested positive in COVID-19-infected patients were urine (17% from COVID-19-positive and 83% from COVID-19-negative patients), followed by serum (tested for *Aspergillus* galactomannan). *Candida* isolation was observed in 27% (21/79) of urine samples and 15% (12/79) of respiratory samples [bronchoalveolar lavage (BAL), tracheal aspirate, and sputum] from COVID-19-positive patients. *Rhizopus arrhizus* and *Rhizopus homothallicus* were isolated from nasal and tissue samples in 6% of COVID-19-positive patients. There was an overall increase in fungal co-isolations during the COVID-19 pandemic (64% in COVID-19-positive and 43% in COVID-19-negative patients), which is a matter of great concern. The correlation of clinical symptomatology and laboratory isolation is important for the diagnosis and effective management of these patients.

## Data Summary

No additional data are required to reproduce the results obtained in this work.

## Introduction

Severe Acute Respiratory Syndrome Coronavirus 2 (SARS-CoV-2), characterized by its high rate of human-to-human transmission through the respiratory route, has been implicated in a diverse spectrum of symptoms associated with Coronavirus Disease 2019 (COVID-19) [1]. The spike protein (S protein) of SARS-CoV-2 primarily interacts with the Angiotensin-Converting Enzyme 2 (ACE2) receptor on human cells, enabling viral entry. The receptor-binding domain of the S protein attaches to ACE2, initiating endocytosis and exposing the virus to endosomal proteases, which subsequently facilitates viral infection in the host. This infection can lead to a range of symptoms, from mild to severe, such as fever, cough, and complications like acute respiratory distress syndrome (ARDS), multiorgan failure etc. In addition to COVID-19 infection, some studies conducted in China have reported an increase in concurrent bacterial, viral, and fungal infections [2].

Lymphocytes are prominently decreased in COVID-19 severe infection due to the excessive utilization of peripheral lymphocytes (hyperactivation of T cells or effect of cytokines on these cells). The possible reason is the adhesion of lymphocytes to respiratory mucosa or endothelium of vessels in respiratory tract. This has been confirmed by single-cell RNA sequencing [3]. There is a severe risk of health deterioration in fungal co-infested COVID-19 patients leading to critical states of patients requiring ventilation, long hospital stay (<= 50 days) and treatment in intensive care units (ICU) [4]. Also in COVID-19 patients, usage of immunotherapy like corticosteroids, IL-6 inhibitors and Janus kinase (JAK) inhibitors are considered to be the predisposing factors in the development of invasive fungal infections (IFI) [5]. The fungal co-infections usually seen are aspergillosis, candidiasis, mucormycosis and cryptococcosis [6].

During the second wave of the COVID-19 pandemic, there was a rise in COVID-19-associated mucormycosis (CAM) in India, which was declared an epidemic by several states and a notifiable disease by the Government of India [7]. Another infection with a high mortality rate is COVID-19-associated pulmonary aspergillosis (CAPA) [8]. This infection had a high incidence rate of 30% in France [9,10]. The association between COVID-19 and the etiological agent *Aspergillus* species in CAPA has been reported in Asia, Europe, South America, and Australia, with more than 100 cases documented [11,13]. Candida co-infection, frequently observed in COVID-19 patients, has been linked to a significantly elevated crude mortality rate. A reduction in inflammatory mediators following immunotherapy has been documented in patients with severe invasive candidiasis. Further studies are required to understand the epidemiology of Invasive Fungal Infections (IFI) complicating COVID-19.

## Aim

To determine the occurrence of fungal isolations and infections among COVID-19 infected patients during the second wave in a tertiary care hospital.

## Methods

This retrospective study was done in Mycology Laboratory, Department of Microbiology, Lady Hardinge Medical College & Associated Hospitals, Delhi (capital of India), during the period of the second wave of COVID-19 infection from 13 March 2021 to 19 June 2021. A total of 460 patients were included in the study, of whom 79 (17%) were COVID-19 positive and 381 (83%) were COVID-19 negative. The clinical specimens, depending on the organ system involvement [including urine, pus, blood, respiratory samples, gastric aspirate, cerebrospinal fluid (CSF), serum and tissues] from patients clinically suspected of having fungal infections (principally invasive pulmonary fungal infection prompting investigation), were processed according to standard protocols [14]. Direct microscopic examination was done on KOH/wet mounts of all the specimens. Samples were inoculated onto two sets of Sabouraud’s dextrose agar tubes with antibiotics and incubated at 37 and 25 °C. The macroscopic and microscopic morphological features of the isolates grown were studied and identified as per standard procedures. While assessing urinary tract infection for patients on catheters, second samples after sterile catheterization were processed to rule out colonization.

Yeast identification was done by microscopy, Chrome agar and Corn Meal agar morphology, along with sugar fermentation and assimilation tests. Mould identification involved Lactophenol cotton blue microscopy of growths, in addition to slide culture and biochemical tests, following standard procedures [14].

Serum galactomannan was done by Platelia Galactomannan Assay (BIORAD). Based on kit literature, the ELISA procedure was done and the optical density of the microplates was read for each well at 450 nm (reference filter of 620/630 nm) within 30 min of the addition of stopping solution.

MucorGenius® real-time PCR was employed for the detection of zygomycetes, and species identification was performed using five genus-specific PN-700 primers in 12 suspected patients (nasal swab – 2, nasal crusts – 4, tissue – 5, nasal discharge – 1). DNA was extracted from the sample according to the standard manufacturer’s instructions. The 10 µl of the extracted nucleic acid was used for PCR. The premix of specific primers of Mucorales was used for real-time PCR and internal control. Positive and negative controls were set up, and the results were observed after 2 hours.

## Statistical analysis

Data were entered into a Microsoft Excel spreadsheet and were checked for any discrepancies. The summarized clinical and laboratory data were presented using tables and graphs. The data were analysed by SPSS (25.0 version, Amork, NY, USA). Shapiro–Wilk test was used to check which variables were following a normal distribution. The data were normally distributed; therefore, inferential statistics were performed using parametric tests. To assess the correlation between categorical variables, the Chi-square test was used. The level of statistical significance was set at a *p*-value of less than 0.05.

## Results

Among a total of 460 patients, the maximum samples received in COVID-19-positive patients (*n*=79) were urine (27%), followed by serum samples (24%), while in COVID-19-negative patients (*n*=381), a maximum of 35% respiratory samples (BAL, tracheal aspirate, sputum) were received, followed by urine (27%), as seen in Table 1. Fungal isolations were observed in urine samples of 33% and 53% of COVID-19-positive and COVID-19-negative patients, respectively. A varied number of isolations were obtained from different samples, including respiratory, nasal, blood, and cerebrospinal fluid (CSF), as shown in Table 1. Additionally, galactomannan was positive in the serum of 22% and 11% of COVID-19-positive and COVID-19-negative patients, respectively.

**Table 1.** Distribution of fungal positivity among COVID-19-positive and COVID-19-negative patients (*N*=460)

Sample	COVID-19-positive patients		COVID-19-negative patients	
	Total (*N*=79)	Positive (*N*=51)	Total (*N*=381)	Positive (*N*=163)
Urine	21 (27 %)	17 (33 %)	101 (27 %)	87 (53 %)
Serum for galactomannan	19 (24 %)	11 (22 %)	33 (9 %)	18 (11 %)
Tissue	11 (14 %)	8 (16 %)	5 (1 %)	1 (1 %)
Respiratory samples(BAL, tracheal aspirate, sputum)	12 (15 %)	8 (16 %)	135 (35 %)	28 (17 %)
Nasal samples(Nasal discharge, nasal crust, nasal swab)	8 (10 %)	4 (8 %)	10 (3 %)	6 (4 %)
Blood	5 (6 %)	2 (4 %)	38 (10 %)	13 (8 %)
Gastric aspirate	1 (1 %)	0 (0 %)	44 (12 %)	4 (2 %)
Cerebrospinal fluid (CSF)	1 (1 %)	1 (2 %)	15 (4 %)	6 (4 %)

Among the 460 patients studied during the period, 79 (17%) were found to be infected with COVID-19, as shown in Table 2. Fungal isolations were observed in 64% (statistically significant with a *p*-value of 0.0004) of COVID-19-positive patients and 43% of COVID-19-negative patients (Table 2).

**Table 2.** Distribution of fungal infections during the second wave of COVID-19 pandemic (*N*=460)

Status	Fungal infection present	Fungal infection absent	Total	***p*-value**	Pearson Chi-square
**COVID-19-positive**	51 (64%)	28 (35%)	79 (17%)	0.0004	12.47
**COVID-19-negative**	163 (43%)	218 (57%)	381 (83%)		
**Total**	214 (47%)	246 (53%)	460		

The fungal positivity mostly observed during the second wave in this study was *Candida* (63% in COVID-19 infected patients and 78% in COVID-19 negative patients), followed by *Aspergillus* (29% in COVID-19 infected patients and 18% in COVID-19 negative patients), as seen in Table 3. Mucormycosis was observed in 6% of COVID-19-positive patients and 1% of COVID-19-negative patients, with *Rhizopus arrhizus* identified as the predominant species (Table 3). Four (33%) *Rhizopus* species were isolated, including three *Rhizopus arrhizus* and one *Rhizopus homothallicus*, from 12 suspected cases of mucormycosis (Table 3). Of these, two *Rhizopus arrhizus* and one *Rhizopus homothallicus* were from COVID-19-positive patients. The RT-PCR test (MucorGenius® real-time PCR) was positive in three patients who were also positive by culture, and in one patient who was negative by culture. The quantitation cycle (*Cq* value) of these positive patients ranged from 20 to 25 with a median *Cq* value of 22.

**Table 3.** Types of fungal isolations based on culture in COVID-19-positive and COVID-19-negative patients (*N*=460)

S.no.	Type of fungal infection	COVID-19-positive patients (*N*=79)	COVID-19-negative patients (*N*=381)
		Total fungal isolation (*N*=51)	Total fungal isolation (*N*=163)
1	Candidiasis	32 (63 %)Species: *C. tropicalis* – 15 (10 – urine, 2 – respiratory sample, 1 – nasal sample, 2 – blood) *C. albicans* – 13 (5 – urine, 3 – respiratory samples, 4 – tissue, 1 – CSF) *C. parapsilosis* – 2 (2 – urine) *C. glabrata* – 1 (1 – respiratory sample: BAL) *C. guillermondii* – 1 (1 – tissue)	127 (78 %)Species: *C. albicans* – 61 [31 – urine, 23 – respiratory samples, 5 – blood, 2 – high vaginal swab (HVS)] *C. tropicalis* – 44 (24 – urine, 13 – respiratory samples, 3 – blood) *C. parapsilosis* – 12 (7 – urine, 4 – blood, 1 – respiratory sample: sputum) *C. glabrata* – 6 (4 – urine, 2 – respiratory samples) *C. krusei* – 3 (2 – urine, 1 – respiratory sample: sputum) *C. lusitanae* – 1 (1 – urine sample)
2	Aspergillosis	15 (29%)Species: *A. fumigatus* – 2 (2 – respiratory samples) *A. flavus* – 2 (2 – nasal tissue sample) 15 serum samples were positive by Galactomannan ELISA (50% [4/8] were also positive by cultures, and 47% [7/15] were serum samples for which cultures were not received)	29 (18%)Species: *A. fumigatus* – 8 (6 – respiratory samples: 4 – BAL, 1 – tracheal aspirate, 2 – sputum) *A. flavus* – 2 (1 – BAL, 1 – ear discharge) *A. niger* – 1 (1 – ear pus) 29 serum samples were positive by Galactomannan ELISA (46% [11/24] were also positive by cultures, and 17% [5/29] were serum samples for which cultures were not received)
3	Mucormycosis	3 (6%)Species: *R. arrhizus* – 2 (1 – tissue from middle meatus, 1 – tissue from middle turbinate) *R. homothallicus* – 1 (1 – nasal crust sample)	1 (1%)Species:*R. arrhizus* – 1 (1 – respiratory sample: sputum)
4	Fusariosis	1 (2%)*F. solani* - 1 (1 – tissue from left turbinate)	0 (0%)
5	Trichophyton infection	0	1 (1%)Species:*T. violaceum –* 1 (1 – nail sample)
6	Paecilomyces infection	0	1 (1%)*P. lilacinus* – 1 (1 –nasal crust sample)
7	*Pneumocystis jiroveci* infection	0	4 (2%)*P. jiroveci* – 4 (2*–*Sputum, 2 *–* Gastric aspirate)

**Fig. 1.**
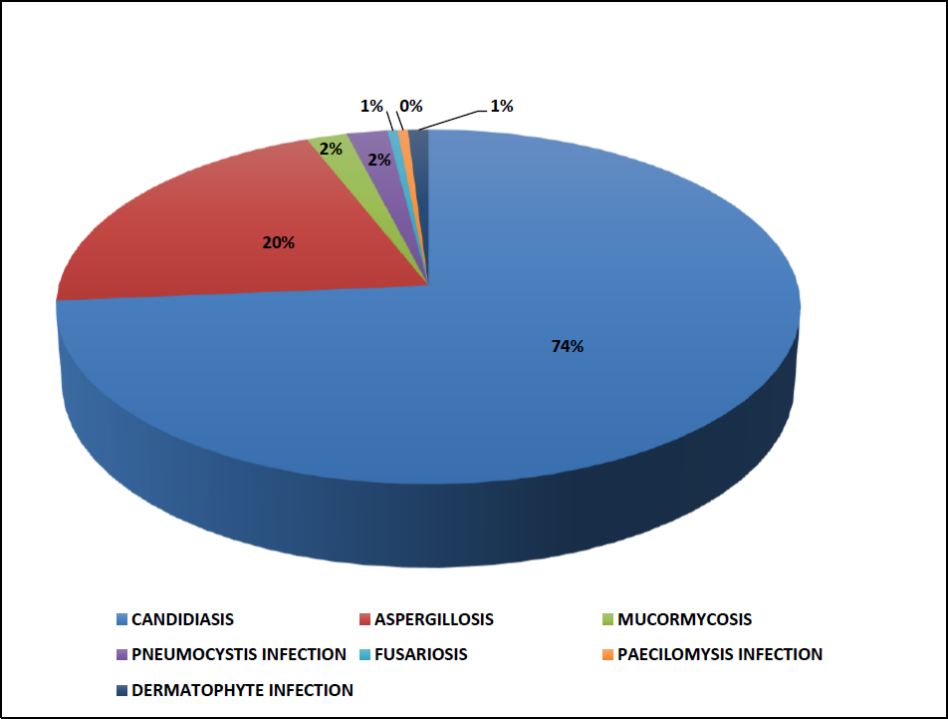
Distribution of fungal positivity (*N*=460)

The maximum fungal positivity among 460 patients was shown to be caused by *Candida* spp. followed by *Aspergillus* spp. (Fig. 1). Among COVID-19-positive patients, urine samples yielded the highest number of *Candida* isolates, predominantly *Candida tropicalis* (Table 3), from patients with a median age range of 8 months to 50 years (Table 4). *C. tropicalis* was in addition isolated in respiratory, blood and nasal samples. *Candida albicans* (*N*=3), *Candida glabrata* (*N*=1) and *C. tropicalis* (*N*=2) were also isolated in the respiratory samples (Table 3), though the significance of their presence in respiratory samples was difficult to assess.

**Table 4.** *Candida* species in COVID-19-positive patients (*N*=32)

Species causing candidiasis	Median age	Gender	Sample	OPD	IPD	Comorbidity/Infection/Symptoms
*C. albicans*	55 years (3–75 years)	Male – 5Female – 8	Urine – 5Respiratory sample – 3Tissue – 4CSF – 1	5	8	Diabetes Mellitus – 86%, Hypertension – 43%, On steroids – 100%
*C. tropicalis*	58 years (1 month– to 80 years)	Male – 6Female – 9	Urine – 10Respiratory sample – 2Nasal sample – 1Blood – 2	1	14	Diabetes Mellitus – 40%, On steroids – 90%
*C. glabrata*	63 years	Male – 1	Respiratory sample – 1	0	1	Pulmonary fibrosis patient on steroids
*C. parapsilosis*	19.5 years (4–35 yrs)	Male – 2	Urine – 2	0	2	On steroids – 50%, Acute Lymphoblastic Leukaemia on Immunosuppressants – 50%
*C. guillermondii*	67 years	Female – 1	Tissue – 1	0	1	On steroids

Among the culture-positive samples from COVID-19-positive patients, *Aspergillus fumigatus* was isolated from respiratory samples, while two isolates of *Aspergillus flavus* were obtained from nasal tissue samples (Table 5). *Rhizopus* spp., predominantly * R. arrhizus*, was isolated from one middle turbinate (nasal) tissue sample and one middle meatus tissue sample, while *R. homothallicus* was isolated from a nasal crust sample (Table 6).

**Table 5.** *Aspergillus* species isolated on culture in COVID-19-positive patients (*N*=4)

Species on fungal culture	Investigations	Sample	Gender	OPD/IPD	Age	Symptoms	Comorbidities
*A. fumigatus*	Microscopy (KOH/wet mount): positiveGalactomannan ELISA: positive	BAL	Male – 2Female – 0	IPD	58 years	Cough, shortness of breath, fever: 100%	Diabetes Mellitus, Prolonged usage of steroids, Hypertension
*A. fumigatus*	Microscopy (KOH/wet mount): negativeGalactomannan ELISA: positive	Sputum	Male	IPD	52 years	Cough, shortness of breath, fever	Diabetes Mellitus, Prolonged usage of steroids
*A. flavus*	Microscopy (KOH/wet mount): negativeGalactomannan ELISA: positive	Nasal tissue sample	Female	IPD	9 years	Sinusitis (Nasal obstruction, headache): 100%	Prolonged usage of steroids
*A. flavus*	Microscopy (KOH mount): negativeGalactomannan ELISA: positive	Nasal tissue sample	Male	OPD	32 years	Sinusitis (Nasal obstruction, headache, runny nose): 100%	Diabetes Mellitus: 100%

**Table 6.** Mucormycosis in COVID-19-positive patients (*N*=3)

Species on fungal culture	Investigations	Gender	Age	Sample	IPD/OPD	Infection/Symptoms	Comorbidities
*R. arrhizus*	Microscopy (KOH mount): negativePCR: positive	Male	37 years	Tissue from middle meatus	ICU	Nasal stuffiness, swollen eyes,eye pain	High dose steroids
*R. arrhizus*	Microscopy(KOH mount): positivePCR: positive	Female	55 years	Tissue from middle turbinate	ICU	Nasal stuffiness	DiabetesHigh dose steroids
*R. homothallicus*	Microscopy(KOH mount): positivePCR: positive	Male	65 years	Nasal crust sample	ICU	Nasal inflammation, nasal discharge, eye pain	Steroid

*Aspergillus fumigatus* (both from respiratory samples) and *Aspergillus flavus* (each 50%) were the species isolated in COVID-19-positive patients. Among these, 75% (3/4) of the patients were admitted to the ICU with a prolonged history of steroid use (Table 3).

## Discussion

The increase in the incidence of fungal co-infestations is a matter of concern. White PL *et al.* stated an incidence rate of 26.7% of COVID-19-associated fungal positivity in 135 cases [15], while a study in China has reported only 5% (5/99) fungal co-infection cases in 99 patients during the culture tests on admission [16]. In our study, suspected COVID-19-positive patients demonstrated a high rate of fungal co-infections (64%), which was statistically significant when compared to 43% in patients without COVID-19 infection.

Variations in the incidence rates of patients with CAPA have been reported, ranging from 4% to 35%, as confirmed by fungal diagnostics and imaging [17]. Zhu X *et al.* in Jiangsu Province of China found 23% fungal co-infections with *Aspergillus* species in one study in 2020 [18]. White PL *et al.* in 2020 found the prevalence of aspergillosis to be 14% in COVID-19 positive patients in a study done in the United Kingdom [19]. In our study, aspergillosis was found to be 29% among COVID-19 positive patients.

Mastrangelo A *et al.* reported that immunosuppression had led to a higher rate of invasive candidiasis during COVID-19 pandemic [20]. The rate of infection by *Candida* has increased by three to eightfold in patients with COVID-19 infection compared to COVID negative patients, according to studies in Brazil and the United States published in 2021 [21,22]. In this study, candidiasis was observed to be 63% among the COVID positive patients, with the most common species isolated being *non-albicans Candida* (60%), as seen in Table 2. Similar results were seen in a study done in Brazil by Martins AC *et al.*, who had reported *non-albicans Candida* as the most common fungal pathogen [23]. However, in a study done by Hughes S *et al.* in the United Kingdom in 2020, *C. albicans* was the most common [24].

Muthu V *et al.* observed that there were 25% cases of mucormycosis (CAM) in the first week of COVID-19 infection in India till 21 June 2021 [25]. Subsequently, COVID-19-associated mucormycosis (CAM) has seen a significant increase, with approximately 20,000 cases reported from various parts of India [26,27]. In this study, 6% of COVID-19-positive patients and 1% of COVID-19-negative patients were found to have mucormycosis (CAM). Based on macroscopic and microscopic morphology, as well as molecular tests, a total of four species were isolated and identified as *Rhizopus arrhizus* (3/4) and *Rhizopus homothallicus* (1/4). RT-PCR results were positive for all these patients, except for one case with a culture-negative nasal crust sample, where RT-PCR aided in the prompt diagnosis.

There have been reports of infections caused by *Fusarium* spp. [28], with 2% of cases involving *Fusarium* reported in our study. Certain fungal infections share symptoms with COVID-19, such as fever, coughing, and difficulty breathing. Among these, COVID-19-associated mucormycosis (CAM) is associated with a high mortality rate (approximately 49%) and increased morbidity [29,30]. In this study, all three confirmed cases of rhinocerebral mucormycosis occurred as co-infections with COVID-19. The infection was characterized by infarction and necrosis of host tissues. All patients had compromised immunity and were critically ill, with each case requiring ICU admission due to the severity of the fungal infection. Martins AC et al. conducted a study in Brazil and reported that in most cases (75%), invasive fungal infection (IFI) was diagnosed after ICU admission [23]. The primary limitation of our study is that it relied solely on clinical and laboratory data (microscopy, culture, and galactomannan positivity). Potential confounders may exist, and extensive follow-up could not be conducted due to limited accessibility and the constraints imposed by the ongoing pandemic.

We conclude that a stringent approach to early clinical suspicion and the implementation of robust diagnostic methods are essential for the timely diagnosis and effective management of suspected IFIs caused by various fungi. Predisposing factors, such as excessive use of steroids and uncontrolled diabetes, must be carefully managed. Thorough examination and evaluation of COVID-19 patients are critical to identifying underlying fungal diseases. Additionally, robust, prospective, multicenter studies are needed to better understand the impact of SARS-CoV-2 on the incidence of fungal isolations progressing to IFIs, particularly in the context of severe disease and immunosuppression in affected patients.

## Abbreviations

ACE2: angiotensin-converting enzyme 2
BAL: bronchoalveolar lavage
CAM: COVID-19-associated mucormycosis
CAPA: coronavirus disease associated pulmonary aspergillosis
COVID-19: coronavirus disease 2019
CSF: cerebrospinal fluid
ICU: intensive care units
IFI: invasive fungal infections
JAK: Janus kinase
